# The complete mitochondrial genome and gene rearrangements in a gall wasp species, *Dryocosmus liui* (Hymenoptera: Cynipoidea: Cynipidae)

**DOI:** 10.7717/peerj.15865

**Published:** 2023-10-03

**Authors:** Cheng-Yuan Su, Dao-Hong Zhu, Yoshihisa Abe, Tatsuya Ide, Zhiwei Liu

**Affiliations:** 1 Laboratory of Insect Behavior and Evolutionary Ecology, College of Life Science and Technology, Central South University of Forestry & Technology (CSUFT), Changsha, Hunan, China; 2 Faculty of Social and Cultural Studies, Kyushu University, Fukuoka, Kyushu, Japan; 3 Department of Zoology, National Museum of Nature and Science, Tsukuba, Ibaraki, Japan; 4 Biological Sciences Department, Eastern Illinois University, Charleston, IL, United States of America

**Keywords:** Mitogenome, Gene rearrangement, Phylogeny, Proctotrupomorpha, Cynipoidea, Cynipidae, *Dryocosmus liui*

## Abstract

Mitochondrial genomes (mitogenomes) have been widely used in comparative and evolutionary genomics, molecular evolution, phylogenetics, and population genetics, but very limited information is available for the family Cynipidae. In this report, we describe the mitogenome of *Dryocosmus liui* Pang, Su et Zhu, providing the first complete mitogenomic data for a cynipid gall wasp species. The mitogenome of *D. liui* is 16,819 bp in length, and contains the typical set of 37 genes. Two control regions were detected, with the second being a perfect inverted repeat of the major portion of the first. Gene rearrangements were found in transfer RNA (tRNA) genes, protein-coding genes (PCGs) and ribosomal RNA (rRNA) genes, compared with the putative ancestral mitogenome. Similar to two other Cynipidae species with mitogenome data available, *D. liui* has a novel tRNA gene cluster *trnL1–trnI–trnL2–trnW–trnM–trnQ* between* nad1* and *nad2*. Phylogenetic analysis based on sequences of PCGs and rRNA genes with *D. liui* included obtained topologies identical to previous studies supporting the a relationship of (Cynipoidea , (Platygastroidea, Proctotrupoidea)) within the monophyletic Proctotrupomorpha and (Cynipidae, Figitidae), Ibaliidae) within the Cynipoidea.

## Introduction

The mitochondrial genome (mitogenome) of most animals is a double-stranded circular DNA molecule and usually includes 13 protein-coding genes (PCGs), 22 transfer RNAs (tRNAs), two ribosomal RNA (rRNA) genes, and a major non-coding sequence called the control region. The size of animal mitogenomes ranges from 13 to 20 kb (Boore, 1999; Cameron, 2014). Given its maternal inheritance, low rate of recombination, relatively high evolutionary rate and conserved gene components, mitogenomes have been widely used in comparative and evolutionary genomics, molecular evolution, phylogenetics, and population genetics (Simon et al. , 2006; Dowton et al. , 2009; Cameron, 2014; Mao, Gibson & Dowton, 2015; Feng et al. , 2020). In recent years, with the development of sequencing technology and the decreased associated costs, there has been a significant increase in the availability of whole mitogenome data, including for hymenopteran insects. Several remarkable features are observed in the hymenopteran mitogenome, including a high A + T content, large variation in substitution rates between lineages, and frequent gene rearrangement (Dowton et al. , 2009; Cameron, 2014; Mao, Gibson & Dowton, 2014; Kahnt et al. , 2015). Most metazoan mitogenomes have rarely experienced gene rearrangements in the course of evolution and such events usually involve only a few tRNA genes (Cameron, 2014). By contrast, gene rearrangements are frequently found in Hymenoptera in tRNA genes and protein-coding genes (Mao, Gibson & Dowton, 2014; Mao, Gibson & Dowton, 2015; Kahnt et al. , 2015; Chen et al. , 2018; Tang et al. , 2019). Rearrangements of mitochondrial genes include transposition, inversion, and inverse transposition (Cameron, 2014). The gene order of the mitogenome contains phylogenetic signals, which may be informative of for phylogenetic relationship among groups (Sankoff et al. , 1992; Dowton et al. , 2009; Tang et al. , 2019).

Cynipidae are a phytophagous group in the superfamily Cynipoidea (Hymenoptera), and its members are well known as gall wasps because they usually induce structurally complex galls on different plant organs, *i.e.,* flowers, leaves, buds, stems, twigs, and roots. It is the second most species-rich group of gall inducers after the gall midge family Cecidomyiidae (Diptera), with roughly 1,400 described species (Ronquist et al. , 2015). In addition to gall formers, the Cynipidae also include phytophagous inquilines, which live inside the galls induced by another species. *Dryocosmus liui* Pang, Su & Zhu (2018) is a recently described species from Hunan Province in central south of China, forming galls on branches of *Castanopsis tibetana* Hance. The genus *Dryocosmus* Giraud is of particular interest among Cynipidae because of its wide distribution, use of host plants belonging to multiple genera in the beech family (Fagaceae), and include a species that is completely parthenogenic (Buffington & Morita, 2009; Zhu et al. , 2015; Pang, Su & Zhu, 2018). In addition, two species of the genus are horticultural pests, *Dryocosmus kuriphilus,* the well known Oriental chestnut gall wasp is a global horticultural pest that causes serious damage to the chestnut industry in its native China as well as in invaded locations (Rieske, 2007; Bosio, Gerbaudo & Piazza, 2010; Zhu et al. , 2015; Martinez-Sañudo et al., 2019), while the recently described *D. zhuili* causes significant damage to *Castanea henryi* in southern China (Zhu et al. , 2015).

The number of complete mitogenome sequences available has increased dramatically in Hymenoptera in recent years, but for Cynipidae, only incomplete mitogenome sequences have been reported for two species, *Synergus* sp. (Tang et al. , 2019) and *Trichagalma acutissimae* (Xue et al. , 2020). In this study, the mitogenome of *D. liui* was sequenced and annotated, providing the first complete mitogenome sequence in the family Cynipidae. The mitogenome structure and gene rearrangements in this lineage were analyzed. In addition, the phylogeny of Proctotrupomorpha was analyzed based on available mitogenome sequences including that of *D. liui* sequenced in the present study.

## Materials and Methods

### Insects and DNA extraction

Galls of *D. liui* were collected on branches of *C. tibetana* from Yanling, Hunan, China (113°93′E, 26°57′N) in March 2016 and March 2020. The galls collected were cage-reared at room temperature in the laboratory of CSUFT. Adults were preserved directly in 100% ethanol at −80 °C within 4 days after emergence until DNA extraction. The gall wasps were washed twice with sterile water to remove surface contamination before DNA extraction. Genomic DNA was extracted from each individual using SDS/proteinase K digestion and phenol–chloroform extraction methods as previously described (Zhu et al. , 2007). The DNA was resuspended in sterile water and stored at 4 °C.

### Mitogenome sequencing and assembly

Short fragments of the *cox1*, *cob*, and *rrnL* genes were amplified using universal insect mitochondrial primers designed by Folmer et al. (1994) and Simon et al. (1994) (Table S1). The PCR products were purified and sequenced directly using the Sanger method at Wuhan Icongene Co, Ltd. (Wuhan, China). Based on the sequences obtained, specific PCR primers (cox1F, cox1R; cobF, cobR and rrnLF, rrnLR) (Table S2) were subsequently designed to amplify the remaining genome by long PCR. The reaction mixture was composed of 1 µL PrimeSTAR^®^ GXL DNA Polymerase (Takara Biomedical Technology Co., Ltd, Dalian, China), 10 µL buffer, 4 µL dNTPs, 1 µL of each primer, and 2 µL of DNA then water was added to give a total volume of 50 µL. The amplification was conducted using a C1000 touch thermal cycler (Bio-Rad, Hercules, CA, USA). The cycling conditions were 98 °C for 3 min, 35 cycles of 98 °C for 10 s, 55–60 °C for 30 s and 68 °C for 10 min. To obtain the complete mitogenome sequence of *D. liui*, the following amplification steps were performed. First, referring to the known mitogenome organization of Cynipoidea (Mao, Gibson & Dowton, 2015; Tang et al. , 2019; Xue et al. , 2020), the primer pairs cox1F/cobR, cobF/rrnLF and rrnLR/cox1R were used to obtain large fragments. A single band of about 9 kb was successfully obtained using the cox1F/cobR primer pairs, but multiple bands appeared when the other two primer pairs were used. Second, because rearrangements of mitochondrial genes are frequently found in Hymenoptera, including transposition, inversion, and inverse transposition, (Mao, Gibson & Dowton, 2014; Mao, Gibson & Dowton, 2015; Kahnt et al. , 2015; Chen et al. , 2018; Tang et al. , 2019), the *D. liui* mitochondrial gene may include rearrangements. Thus, we used cobF/rrnLR and rrnLF/cox1R primer pairs to amplify the rest of the genome fragments, and were able to obtain about 4 kb and 3 kb target bands, respectively. Finally, to verify the accuracy of the results, the full-length sequence of the *D. liui* mitogenome was amplified using cox1F/rrnLR and cobR/rrnLF primer pairs, and single target bands of about 12 kb and 14 kb were obtained, respectively (Fig. S1).

Those PCR products were subsequently purified using a TaKaRa MiniBEST Agarose Gel DNA Extraction Kit Ver.4.0 (Takara Biomedical Technology Co.). The primer walking method was employed to determine the sequence for each long PCR product using an ABI 3730XL DNA sequencer (Applied Biosystems, Foster City, CA, USA) at Wuhan Icongene Co, Ltd. (for sequencing primers see Tables S2–S3). Raw sequences were assembled into contigs in ChromasPro Ver 1.33 (Technelysium Ltd., Tewantin, Australia), then checked and corrected manually. The sequencing results of the long PCR products obtained in the third step described above were completely consistent with those in the first and second steps, validating the reliability of the amplification strategy used in this study.

### Mitogenome annotation and genome feature analysis

The PCGs were identified by the ORF Finder in NCBI (http://www.ncbi.nlm.nih.gov), by specifying the invertebrate mitochondrial genetic code and were confirmed by alignment with homologous genes from closely related species in Geneious 10.1.3. The start and stop codons of some genes were corrected according to the boundaries of tRNA genes and through alignment with other hymenopteran mitogenome sequences. The tRNA genes were initially identified using tRNA-scan SE 1.21 (http://lowelab.ucsc.edu/tRNAscan-SE/), with the parameters: source = “Mito/Chloromast” and genetic code = “Invertebrate Mito”. Twenty-one of the 22 typical animal mitochondrial tRNA genes were identified. The remaining one tRNA and two rRNA genes were identified by the MITOS WebServer using the invertebrate mitochondrial genetic code (http://mitos.bioinf.uni-leipzig.de/index.py). The rRNA genes were confirmed by sequence comparison with published hymenopteran mitochondrial rRNA sequences. The annotated complete mitogenome sequence of *D. liui* has been submitted to GenBank with the accession number MW368384.

The nucleotide composition of the mitogenome was calculated using MEGA 7.0. We also measured the AT- and GC-skews using the following formulas: AT-skew = (A − T)/(A + T), GC-skew = (G − C)/(G + C), for whole mitogenome sequences and 13 PCGs. The tandem repeats in the two control regions were predicted using the Tandem Repeats Finder available online (https://tandem.bu.edu/trf/trf.html).

### Phylogenetic analyses

For the phylogenetic analyses, the mitogenome sequences of 19 species of Proctotrupomorpha, including five species of Cynipoidea, were included and additional four species of the sister clade Ichneumonomorpha were used as outgroups (Table S4). Nucleotide sequences for each of the two rRNA genes and the 13 protein-coding genes were imported into separate files using MEGA7.0.

The two rRNA genes were individually aligned using the MAFFT 7.0 online server with a Q-INS-i strategy (Katoh & Standley, 2013). Each protein-coding gene was aligned individually using codon-based multiple alignments and the MAFFT algorithm within the TranslatorX server. A nucleotide alignment was inferred from the amino acid alignment. All alignments were subsequently checked and corrected manually in Geneious 10.1.3 for quality control. Individual gene alignments were concatenated using SequenceMatrix prior to phylogenetic analysis. Alignments were concatenated as two datasets: (1) the PCG123+2rRNA matrix, including all three codon positions of PCGs and two rRNA genes; and (2) the PCG12+2rRNA matrix, including the first and second codon positions of PCGs and two rRNA genes. Maximum likelihood, Bayesian and PhyloBayes approaches were employed to infer phylogenetic trees using MrBayes v. 3.2.7a, IQ-Tree 2.1.1, and PhyloBayes MPI 1.7b, respectively, *via* the online CIPRES Science Gateway portal (Miller, Pfeiffer & Schwartz, 2010). For Bayesian analyses, the best partitioning schemes and corresponding nucleotide substitution models were determined with PartitionFinder2 v.2.1.1 *via* the online CIPRES Science gateway portal, and two independent runs were performed with the default priors and MCMC parameters except that the MCMC runs comprised 10 million generations sampled at every 1,000 generations with 25% burn-in time. For maximum likelihood analyses, a total of 10,000 bootstrap replicates were obtained with the auto model applied to all partitions. For PhyloBayes analyses, phylogenetic trees were inferred from the datasets with the CAT + GTR model.

## Results and Discussion

### Genome structure and nucleotide composition

**Figure 1:**
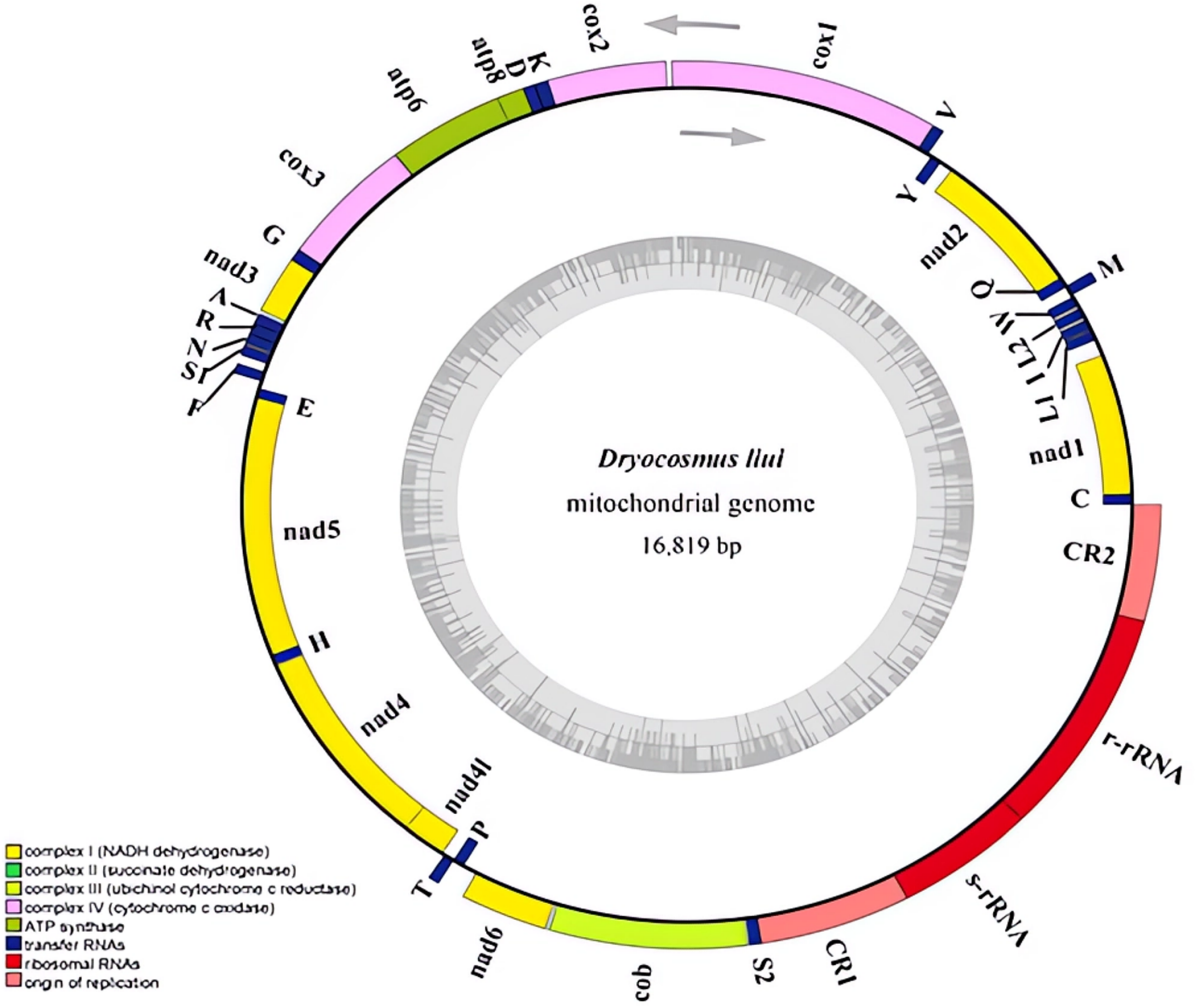
Mitochondrial genome of *Dryocosmus liui* sequenced in this study. Genes outside the circle are encoded by the major coding strand, and inside genes are encoded by the minor strand. Abbreviations of gene names are: atp6 and atp8 for atp-synthase subunits 6 and 8, cox1-3 for cytochrome oxidase subunits 1–3, cob for cytochrome b, nad1-6 and nad4L for NADH dehydrogenase subunits 1–6 and 4 L, l-rRNA and s-rRNA for arge and small rRNA subunits. tRNA genes are indicated with their one-letter corresponding amino acids. CR for control region. The GC content is plotted using a black sliding window.

The complete mitogenome of *D. liui* was sequenced, and its length was 16,819 bp (GenBank accession number: MW368384). The mitogenome contained the typical gene repertoire of 13 protein-coding genes (*nad1-6*, *nad4L*, *cox1-3*, *atp6*, *atp8*, and *cob*), two rRNA genes (*rrnL*, *rrnS*) and 22 tRNA genes; among these, 22 genes were found on the majority strand (J-strand) and 15 genes were on the minority strand(N-strand) (Fig. 1 and Table 1). A total of 660 bp of intergenic nucleotides ranging from 2 to 104 bp were found in 21 locations. The longest intergenic spacer (104 bp) was found between *nad1* and *trnL*. There were 112 overlapping nucleotides dispersed in eight locations, *i.e., trnM*-*trnQ*, *trnQ*-*nad2*, *cox2*-*trnk*, *atp8*-*atp6*, *trnA-trnR*, *trnR-trnN*, *trnE-nad5*, and *nad4*-*nad4l*, and their length ranged from 1 to 41 bp (Table 1). The whole mitogenome had an overall base composition of 41.9% A, 41.8% T, 8.1% G, and 8.2% C, with a high AT bias of 83.7% (Table S5), as reported for most hymenopteran insects (Chen et al. , 2018). The mitogenome has a positive AT-skew value (0.0016) and negative GC-skew value (−0.0095), biased for the A and C nucleotides (Table S5).

**Table 1:** Annotation of the *Dryocosmus liui* mitochondrial genome.

Gene	Position (bp)	Size (bp)	Strand^*^	Intergenic nucleotides	Anti or Start codon	Stop codon	A + T%
*trnC*	1–64	64	−	2	GCA		89.1
*nad1*	67–984	918	−	104	ATA	TAA	80.6
*trnL1*	1089–1115	67	−	6	TAG		90.1
*trnI*	1162–1231	70	−	14	GAT		81.4
*trnL2*	1246–1315	70	−	14	TAA		87.1
*trnW*	1330–1398	69	−	13	TCA		91.3
*trnM*	1412–1477	66	+	−5	CAT		89.4
*trnQ*	1473–1541	69	−	−41	TTG		88.4
*nad2*	1501–2499	999	−	81	ATG	TAA	89.8
*trnY*	2581–2649	69	−	25	GTA		89.8
*trnV*	2675–2738	64	+	6	TAC		92.2
*cox1*	2745–4292	1548	+	31	ATG	TAA	74.7
*cox2*	4324–5022	699	+	−20	ATA	TAA	79.4
*trnK*	5003–5081	79	+	0	TTT		88.6
*trnD*	5082–5148	67	+	0	GTC		92.5
*atp8*	5149–5307	159	+	−7	ATT	TAG	87.4
*atp6*	5301–5975	675	+	5	ATG	TAA	82.3
*cox3*	5981–6772	792	+	0	ATG	TAA	77.7
*trnG*	6773–6844	72	+	0	TCC		91.7
*nad3*	6845–7195	351	+	21	ATT	TAA	85.2
*trnA*	7217–7281	65	+	−2	TGC		86.2
*trnR*	7280–7348	69	+	−1	TCG		85.5
*trnN*	7348–7414	67	+	7	GTT		85.1
*trnS1*	7422–7487	66	+	54	TCT		87.9
*trnF*	7542–7604	63	+	47	GAA		88.9
*trnE*	7652–7716	65	−	−29	TTC		95.4
*nad5*	7688–9307	1620	−	90	ATT	TAA	84.0
*trnH*	9398–9461	64	−	9	GTG		79.7
*nad4*	9471–10790	1320	−	−7	ATG	TAA	83.9
*nad4l*	10784–11056	273	−	16	ATT	TAA	83.9
*trnT*	11073–11137	65	+	0	TGT		87.7
*trnP*	11138–11207	70	−	88	TGG		82.9
*nad6*	11296–11805	510	+	16	ATT	TAG	89.0
*cob*	11823–12956	1134	+	11	ATG	TAA	77.5
*trnS2*	12968–13035	68	+	0	TGA		85.3
CR1	13036–13907	872		0			82.6
*rrnS*	13908–14715	808	+	0			90.5
*rrnL*	14716–16106	1391	+	0			89.4
CR2	16107–16819	713					80.5

**Notes.**

* + indicates the gene is coded on majority strand while −indicates the gene is coded on minority strand.

### PCG, tRNA, and rRNA genes

In the *D. liui* mitogenome, eight of the 13 protein-coding genes were located on the majority strand, while the other five protein-coding genes were encoded by the minority strand. The average A + T content of the 13 protein-coding genes was 81.7%, and the highest was 89.8% for *nad2* (Table 1, Table S5). All PCGs started with the standard ATN codons, as in most other insect mitogenomes. There were six genes (*nad2*, *cox1*, *atp6*, *cox3*, *nad4*, and *cob*) starting with ATG, five genes (*atp8*, *nad3*, *nad5*, *nad4l*, and *nad6*) starting with ATT, and two genes (*nadl* and *cox2*) starting with ATA. Most of the PCGs terminated with a TAA stop codon, while *atp8* and *nad6* ended with a TAG codon (Table 1).

The 22 tRNA genes of *D. liui* were spread across nine clusters; 12 were on the majority strand while 10 were located on the minority strand. The tRNA genes ranged from 63 bp (*trnF*) to 79 bp (*trnK*) (Table 1). All the tRNA genes could be folded into the typical cloverleaf secondary structure, except for *trnT* which lacked a complete T*ψ*C-loop (Fig. S2). In most insects, the length of the acceptor arm (7 bp) and of the anticodon arm (stem: 5 bp, loop: 7 bp) is highly conserved across tRNAs whereas the DHU- and TψC-arms are quite variable in size (Kahnt et al. , 2015). In the *D. liui* mitogenome, the anticodon arms of *trnA* and *trnM* were 4 bp in stems and 9 bp in loops. The stem of the DHU-arm ranged from 2 to 4 bp and the loop from 4 to 10 bp; the stem of the TψC-arm varied between 3 and 5 bp; and the loop between 3 and 9 bp (but *trnk* is 16 bp). Sixteen mismatched base pairs were detected in the tRNAs of *D. liui*, all of which were G-U or U-U pairs.

The positions of the two rRNAs appear to be highly conserved across Hymenoptera (Mao, Gibson & Dowton, 2015). However, *D. liui* is different from most hymenopteran insects: the large and small rRNA genes(*rrnL* and *rrnS*), are next to each other between two large non-coding regions on the minority strand; the length of the *rrnS* gene was 808 bp, with an A + T content of 90.5%, while the *rrnL* gene was 1391 bp with an A + T content of 89.4% (Table 1).

### Control region

**Figure 2:**
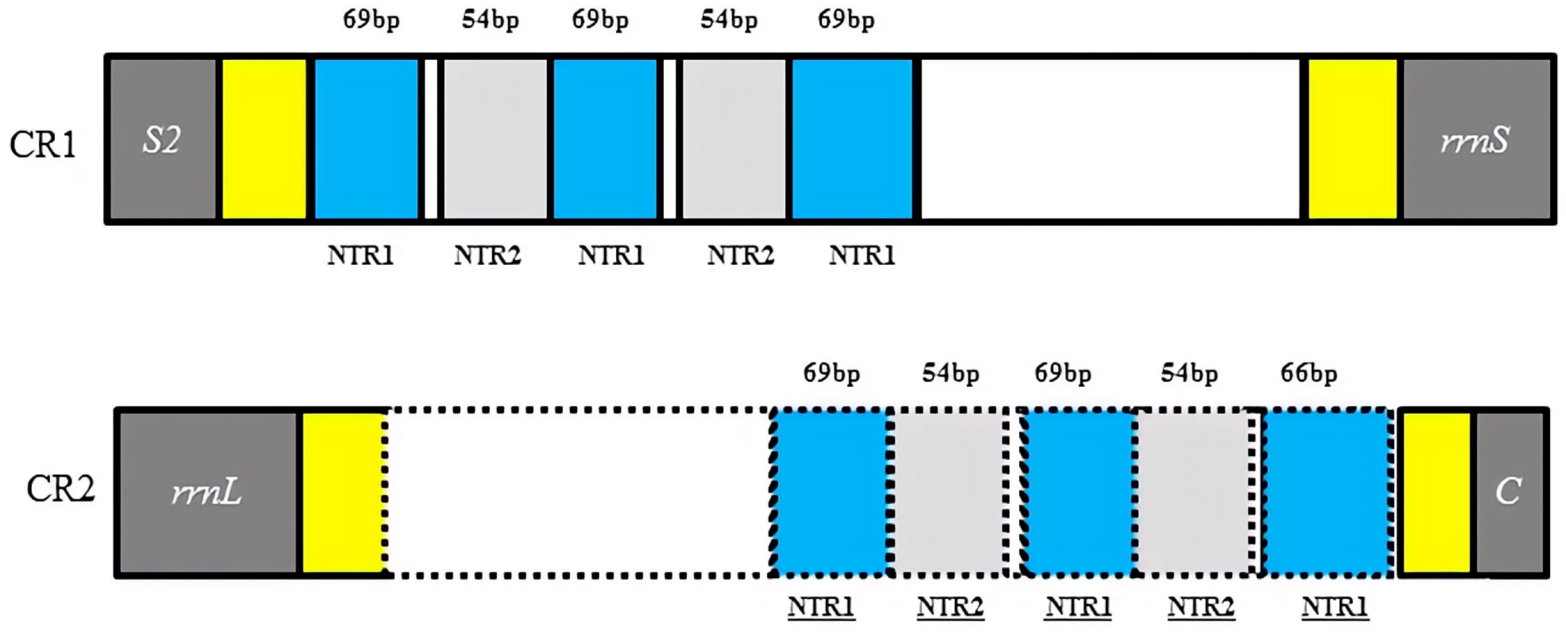
Structures of the two control regions in *Dryocosmus liui* mitochondrial genome. NTR indicates nontandem repeat. Yellow shows A + T rich regions. Within the dotted line in CR2 is the inverted, duplicated sequence of CR1.

Two copies of control regions (A + T rich regions) were detected in the *D. liui* mitogenome and were flanked by *trnS2* and *rrnS* (872 bp in length), and *rrnL* and *trnC* (713 bp in length), respectively (Table 1). In the first copy, three 69 bp and two 54 bp nontandem repeat units, a 122 bp A + T rich region (AT% = 94.3%) and a 129 bp A + T rich region (AT% = 90.6%) were present. The second copy was a perfect inverted repeat of the major portion of the first copy, including three 69 bp and two 54 bp repeat units, a 65 bp A + T rich region (AT% = 95.5%), and a 29 bp A + T rich region (AT% = 82.8%) (Fig. 2). Duplicated control regions (or multiple control regions) are not universal among insect species, and are mainly found in barklice, thrips, and wasps (Shao & Barker, 2003; Mao, Gibson & Dowton, 2015; Yoshizawa et al., 2018; Tyagi et al. , 2020). Among Hymenoptera, this phenomenon has been found in *Orthogonalys pulchella* (Trigonalyoidea: Trigonalyidae), *Ceraphron* sp. (Ceraphronoidea: Ceraphronidae) (Mao, Gibson & Dowton, 2014), *Ibalia leucospoides* (Cynipoidea: Ibaliidae) (Mao, Gibson & Dowton, 2015), *Alloxysta* sp. (Cynipoidea: Figitidae) (Tang et al. , 2019), and *Aphidius gifuensis* (Ichneumonoidea: Aphidiinae) (Feng et al. , 2020). To our knowledge, the inverted, duplicated control regions have been found only in Cynipoidea, *i.e., D. liui* (this study) and *Ibalia leucospoides* (Mao, Gibson & Dowton, 2015). It is proposed that the presence of two control regions may be advantageous and be maintained either through stabilizing selection or through gene conversion (Eberhard, Wright & Bermingham, 2001). Furthermore, a polyT stretch at the 5′ end of the control region and a [TA(A)]n-like stretch following the polyT stretch could be found in the two copies of the control region, and have been proposed as initiation sites for replication and transcription of the mitochondrial genome (Wei et al., 2010).

### Gene arrangement

**Figure 3:**
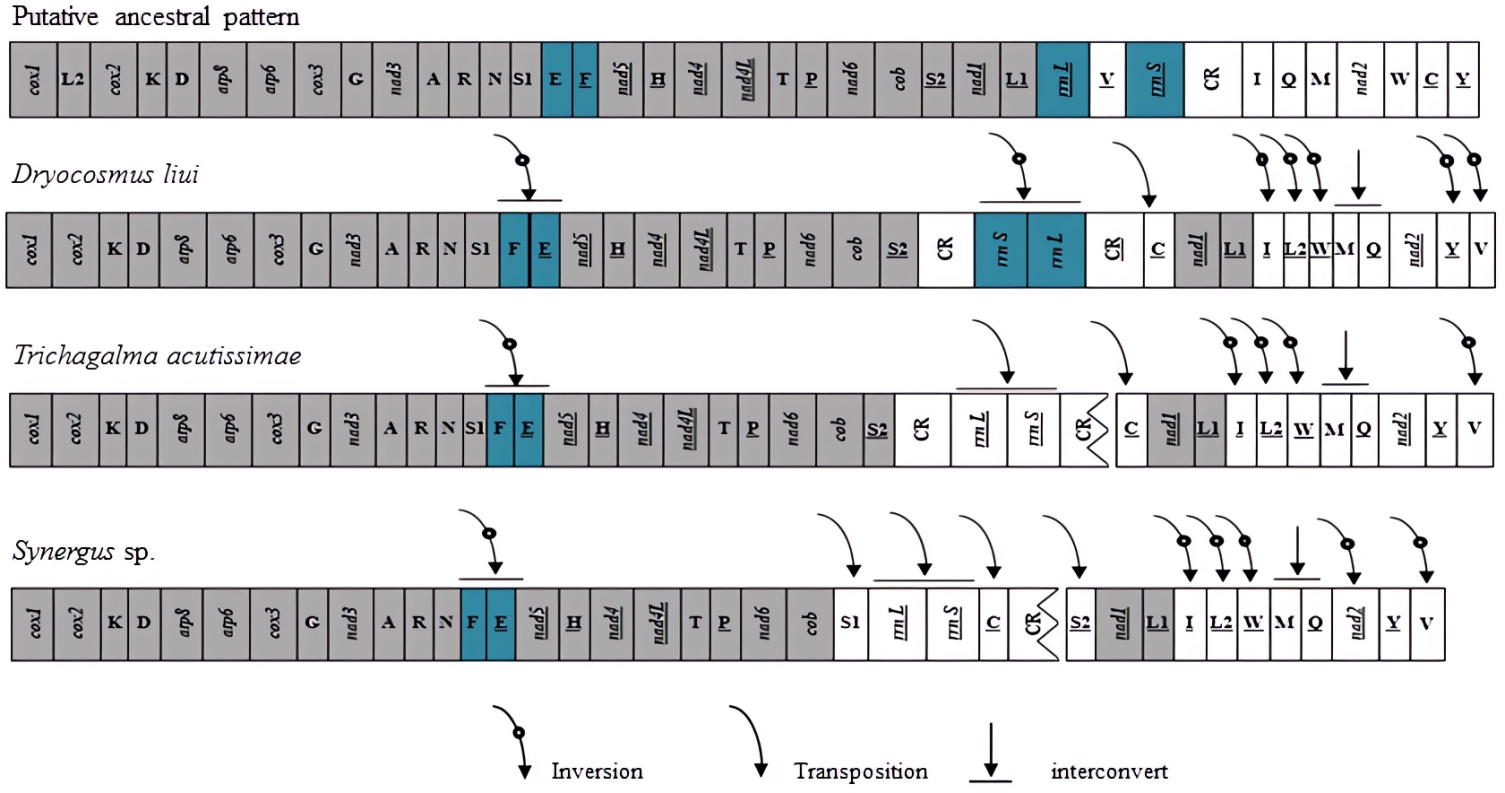
Mitochondrial genome organization and gene rearrangement in Cynipidae compared with the ancestral type of the insect mitochondrial genome. The gene order is linearized for easy view. The gene blocks with inversion are shown in dark blue, while the conserved gene blocks are showed in grey.

Gene rearrangements have been found not only in tRNA genes but also in protein-coding genes in several species of Hymenoptera (Mao, Gibson & Dowton, 2014; Mao, Gibson & Dowton, 2015; Kahnt et al. , 2015; Chen et al. , 2018; Tang et al. , 2019). In these mitogenomes, the gene clusters between *cox2*–*atp8*, *nad3*–*nad5*, *nad2*–*cox1*, and control region–*nad2* have been identified as the most frequently rearranged regions (Mao, Gibson & Dowton, 2014; Wei et al. , 2014; Chen et al. , 2018). When compared with the putative ancestral pattern of the pancrustacean/hymenopteran mitogenome (Cook, 2005; Mao et al. , 2012), rearrangement events of the *D. liui* mitogenome were found to be mainly on the gene clusters between *nad3*–*nad5*, *nad2*–*cox1*, and control region–*nad2*, involving PCG, tRNA, and rRNA genes, respectively (Fig. 3). There are nine tRNA gene rearrangements relative to their ancestral positions: *trnE* and *trnF* have inverted and swapped positions; *trnC* has moved to a position upstream of *nad1*; *trnV* has inverted and moved to the *nad2*–*cox1* gene junction; *trnQ* has swapped position with *trnM*; and *trnL2* and *trnW* have inverted and changed positions, with inverted *trnI* forming a novel tRNA gene cluster *trnL1–trnI–trnL2–trnW–trnM–trnQ* between *nad1* and *nad2*. The protein-coding gene *nad2* is inverted in *D. liui*, as was reported for *Ibalia leucospoides* (Cynipoidea: Ibaliidae) (Mao, Gibson & Dowton, 2015). Gene rearrangement was also found in rRNA genes in the *D. liui* mitogenome. The two rRNA genes have inverted and moved into the *cob–nad1* junction, flanked by the control region and an inverted, duplicated control region. Inverted rRNA genes have been reported in other insect orders, the Psocodea and Thysanoptera (Shao & Barker, 2003; Covacin et al. , 2006; Cameron, Johnson & Whiting, 2007; Cameron et al., 2011). However, rearrangements of the rRNA genes are rare in Hymenoptera, only occurring in one species, *Megalyra* sp. (Megalyroidea) (Mao, Gibson & Dowton, 2014; Tang et al. , 2019), making what is observed in the mitogenome of *D. liui* regarding the rRNA genes particularly interesting.

We also analyzed the mitochondrial gene rearrangements of two other Cynipidae species, *Trichagalma acutissimae* (MN928529, Xue et al. , 2020) and *Synergus* sp. (MG923514, Tang et al. , 2019) based on incomplete mitogenome sequences obtained from the Genbank. There are different mitochondrial gene rearrangement events among the three gall wasp species relative to the ancestral hymenopteran (Fig. 3), but they have four obvious common characteristics: *trnE* and *trnF* have inverted and swapped positions; *rrnS* and *rrnL* genes have moved into the *cob–nad1* junction; a novel tRNA gene cluster *trnL1–trnI–trnL2–trnW–trnM–trnQ* has been formed between *nad1* and *nad2*; and *trnV* has inverted and moved to the *nad2*–*cox1* gene junction. Whether these events are common among Cynipidae species remains unclear, due to the scarcity of mitogenome sequence data for other species of the family.

### Phylogenetic analysis

**Figure 4:**
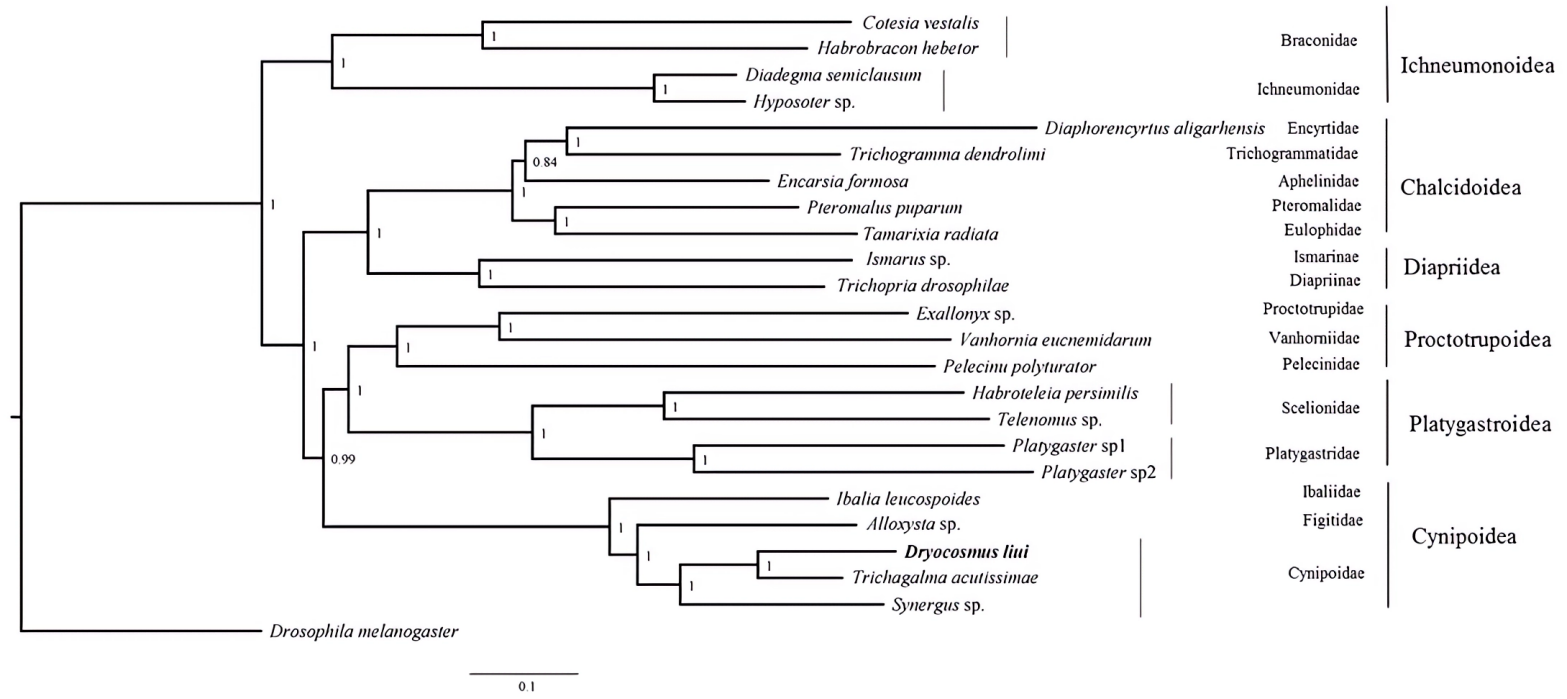
Bayesian analysis of the MAFFT alignment dataset were inferred from the datasets PCG123 +2rRNA. Posterior probabilities are shown at each node.

Phylogenetic analyses were performed using available mitogenome sequences of 19 Proctotrupomorpha. Two datasets (PCG123 + 2rRNA and PCG12 + 2rRNA) were analyzed using the MAFFT alignment approach and three phylogenetic approaches (Bayesian inference, PhyloBayes and Maximum likelihood). Different analytical approaches and datasets did not affect the topology, but did affect the level of node support (Fig. 4 and Figs. S3–S7).

Within the Proctotrupomorpha, most of the recent molecular analyses have generally recovered a sister relationship for Cynipoidea and Platygastroidea (Castro & Dowton, 2006; Dowton & Austin, 2001; Heraty et al. , 2011; Klopfstein et al. , 2013; Tang et al. , 2019). In this study, the five superfamilies clustered into two branches, namely Chalcidoidea + Diaprioidea and Proctotrupoidea + Platygastroidea + Cynipoidea. It is strongly suggested that Cynipoidea are closely related to Platygastroidea and Proctotrupoidea, with high nodal support (BPP = 1) (Fig. 4). The support values were higher in the analyses using the PCG123 + 2rRNA dataset and BI phylogenetic approach, which may suggest that the inclusion of 3rd codon positions and rRNA genes for Bayesian analysis was more suitable for making inferences regarding this relationship, as discussed by Tang et al. (2019). The relationship within Cynipoidea based on limited mitogenome data is consistent with multiple phylogenetic analyses based on various types of data with more extensive sampling (Ronquist, 1995; Ronquist, 1999; Ronquist et al. , 2015; Liljeblad & Ronquist, 1998; Rokas et al. , 2003; Blaimer et al. , 2020). It would be highly desirable to sample more extensively in future projects to reconstruct Cynipoidea phylogeny based on mitogenome independently and in combination with other types of data.

## Conclusions

In summary, the mitogenome contained the typical gene repertoire of 13 protein-coding genes, two rRNA genes and 22 tRNA genes. Inverted and duplicated control regions have been found which may be advantageous and be maintained either through stabilizing selection or through gene conversion. Rearrangement events of the *D. liui* mitogenome were found to be mainly on the gene clusters between *nad3*–*nad5*, *nad2*–*cox1*, and control region–*nad2*, involving PCG, tRNA, and rRNA genes, respectively. It is obvious that ,the mitochondrial genome is a powerful molecular markers for phylogenetic analyses.

## Supplemental Information

10.7717/peerj.15865/supp-1 Figure S1 PCR products of themitochondrial genome fragment using different primer pairs : 1 (cox1F / cobR); 2 (cobF /rrnLF); 3 (rrnLR /cox1R); 4 (cobF /rrnLR) ;5 (rrnLF /cox1R); 6 (cox1F /rrnLR) and 7 (cobR / rrnLF), M for 1kb DNA Marker

10.7717/peerj.15865/supp-2 Figure S2 Predicted folding pattern for tRNAs of Dyocosmus liui mitochondrial genome

10.7717/peerj.15865/supp-3 Figure S3 Bayesian analysis of the MAFFT alignment dataset were inferred from the datasets PCG12+2RNA Posterior probabilities are shown at each node.

10.7717/peerj.15865/supp-4 Figure S4 Maximum Likelihood tree were inferred from the datasets PCG123+2RNA using IQ-tree with the auto model Posterior Probability /approximate likelihood ratio test are shown at each node.

10.7717/peerj.15865/supp-5 Figure S5 Maximum Likelihood tree were inferred from the datasets PCG12+2RNA using IQ-tree with the auto model Posterior Probability /approximate likelihood ratio test are shown at each node.

10.7717/peerj.15865/supp-6 Figure S6 Phylogenetic tree were inferred from the datasets PCG123+2RNA using PhyloBayes with the CAT+GTRmodel Posterior probabilities are shown at each node.

10.7717/peerj.15865/supp-7 Figure S7 Phylogenetic tree were inferred from the datasets PCG12+2RNA using PhyloBayes with the CAT+GTRmodel Posterior probabilities are shown at each node.

10.7717/peerj.15865/supp-8 Table S1 List of universal insect mitochondrial short fragments of the cox1, cob, and rrnL genes primers used for long PCR primer developments

10.7717/peerj.15865/supp-9 Table S2 List of PCR primers and sequencing primers used to determine the sequence for each long PCR product (cox1F / cobR, cobF / rrnLR and rrnLF / cox1R)

10.7717/peerj.15865/supp-10 Table S3 List of PCR primers and sequencing primers used to verify the accuracy of the results using cox1F / rrnLR and cobR / rrnLF primer pairs

10.7717/peerj.15865/supp-11 Table S4 Summary of taxonomic groups used in this study

10.7717/peerj.15865/supp-12 Table S5 Genome feature analysis

## Additional Information and Declarations

### Data Availability

The following information was supplied regarding data availability: Data is available at GenBank: MW368384.
