# Supplementary figures and images for: The complete mitochondrial genome and gene rearrangements in a gall wasp species, Dryocosmus liui (Hymenoptera: Cynipoidea: Cynipidae)
Source: PeerJ. 2023 Oct 3;11:e15865. doi: 10.7717/peerj.15865 (PMC10557937; doi:10.7717/peerj.15865)

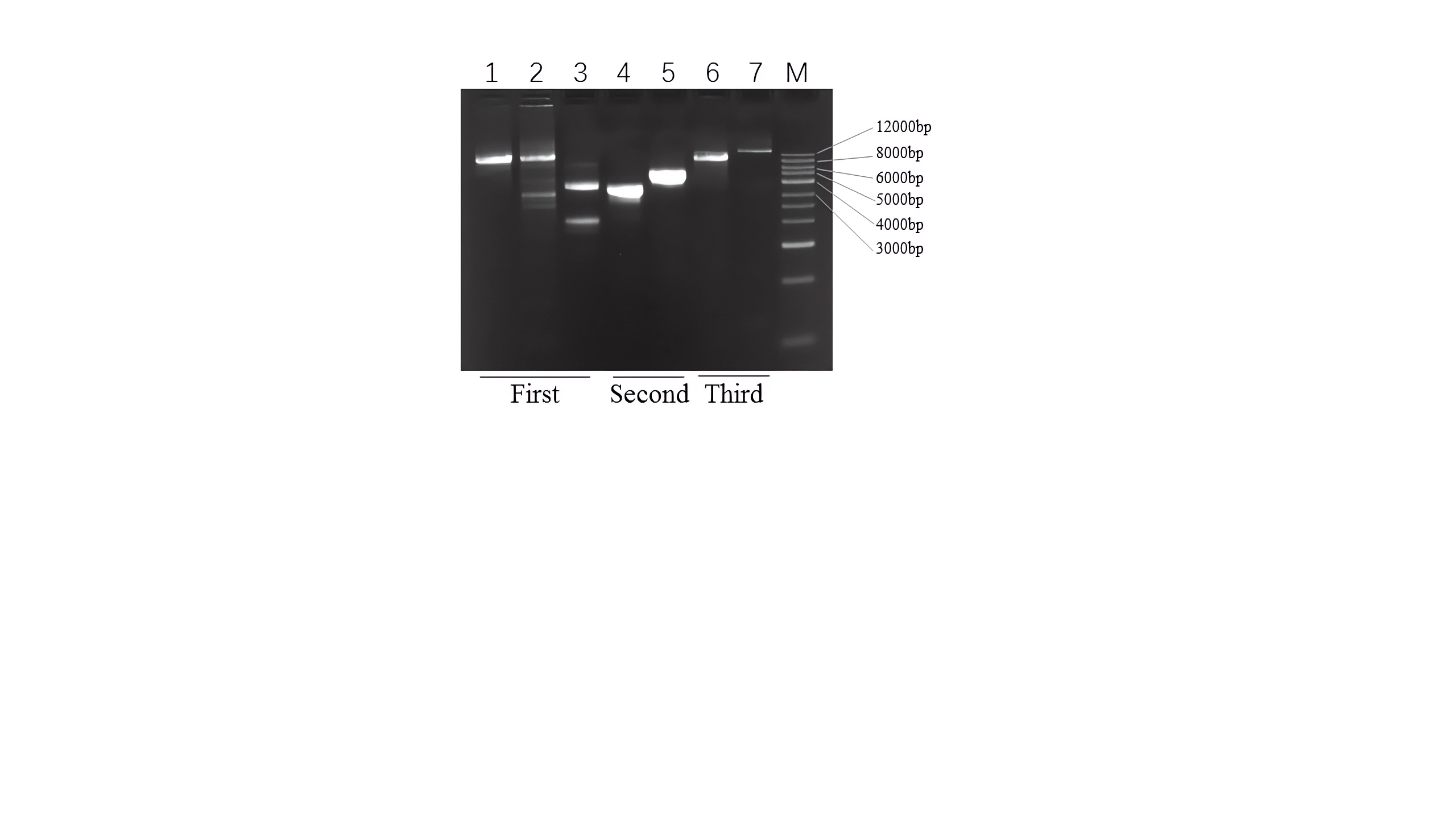

Supplement: Figure S1 [file peerj-11-15865-s001.png]

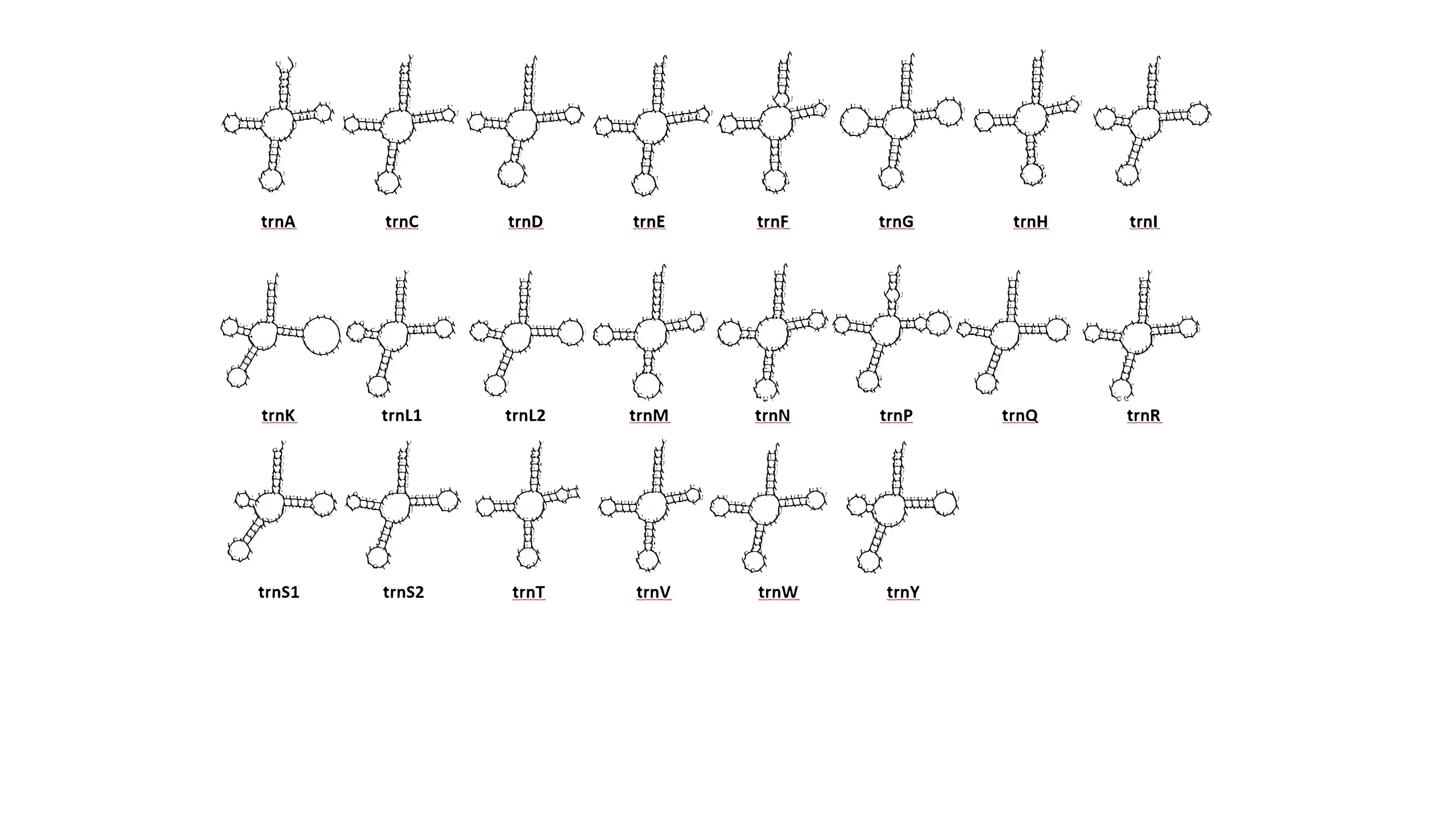

Supplement: Figure S2 [file peerj-11-15865-s002.png]

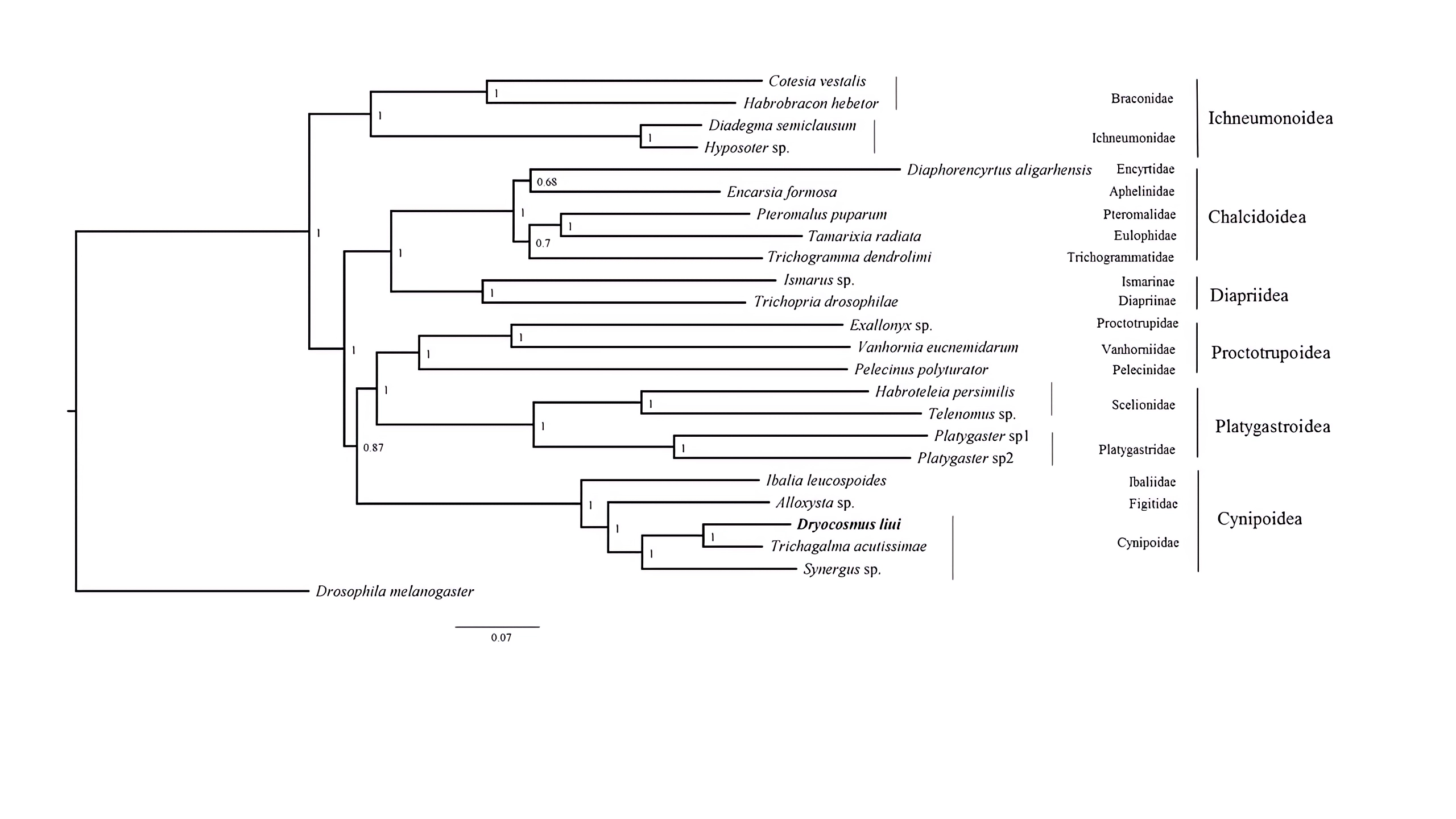

Supplement: Figure S3 — Posterior probabilities are shown at each node. [file peerj-11-15865-s003.png]

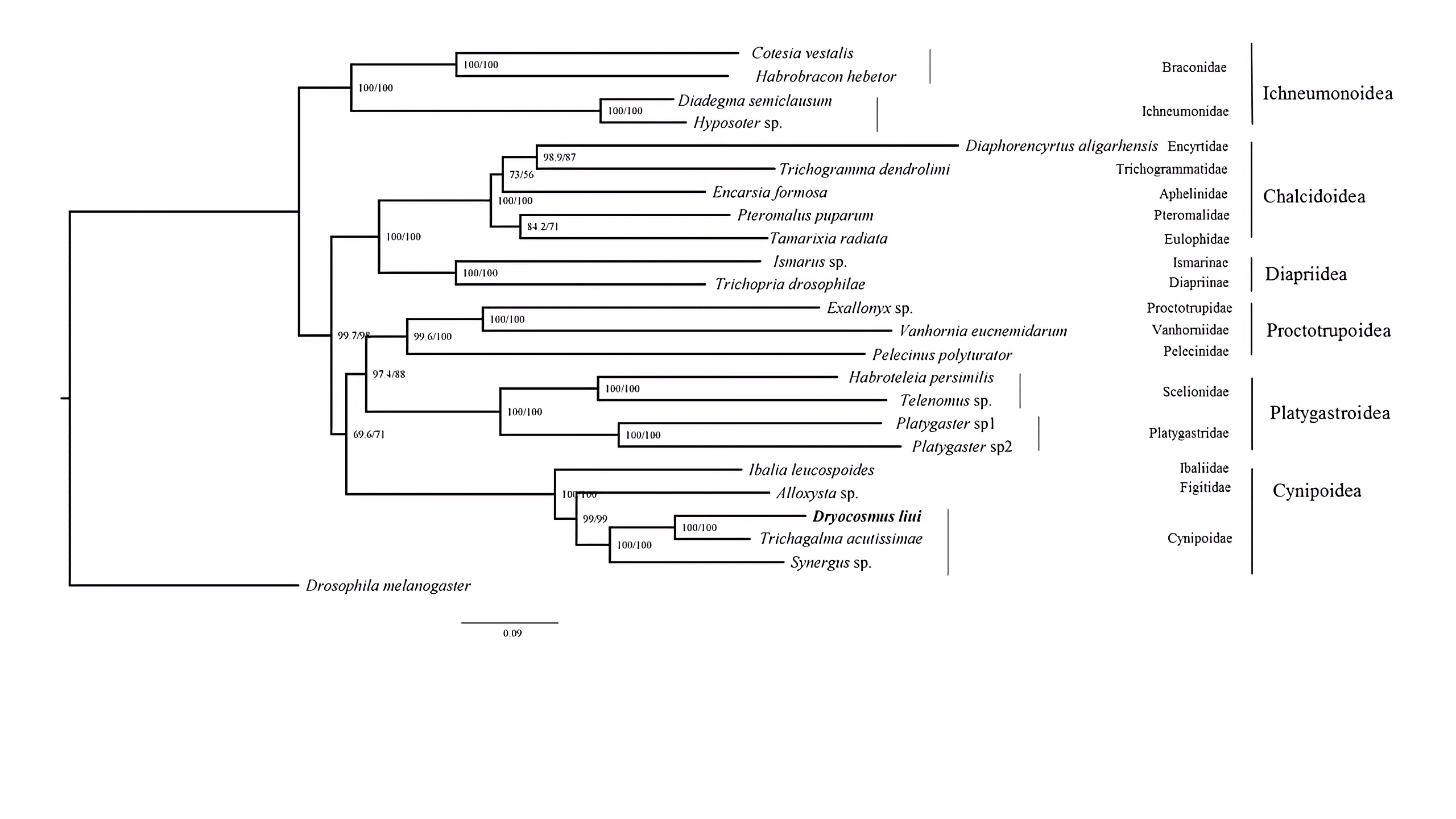

Supplement: Figure S4 — Posterior Probability /approximate likelihood ratio test are shown at each node. [file peerj-11-15865-s004.png]

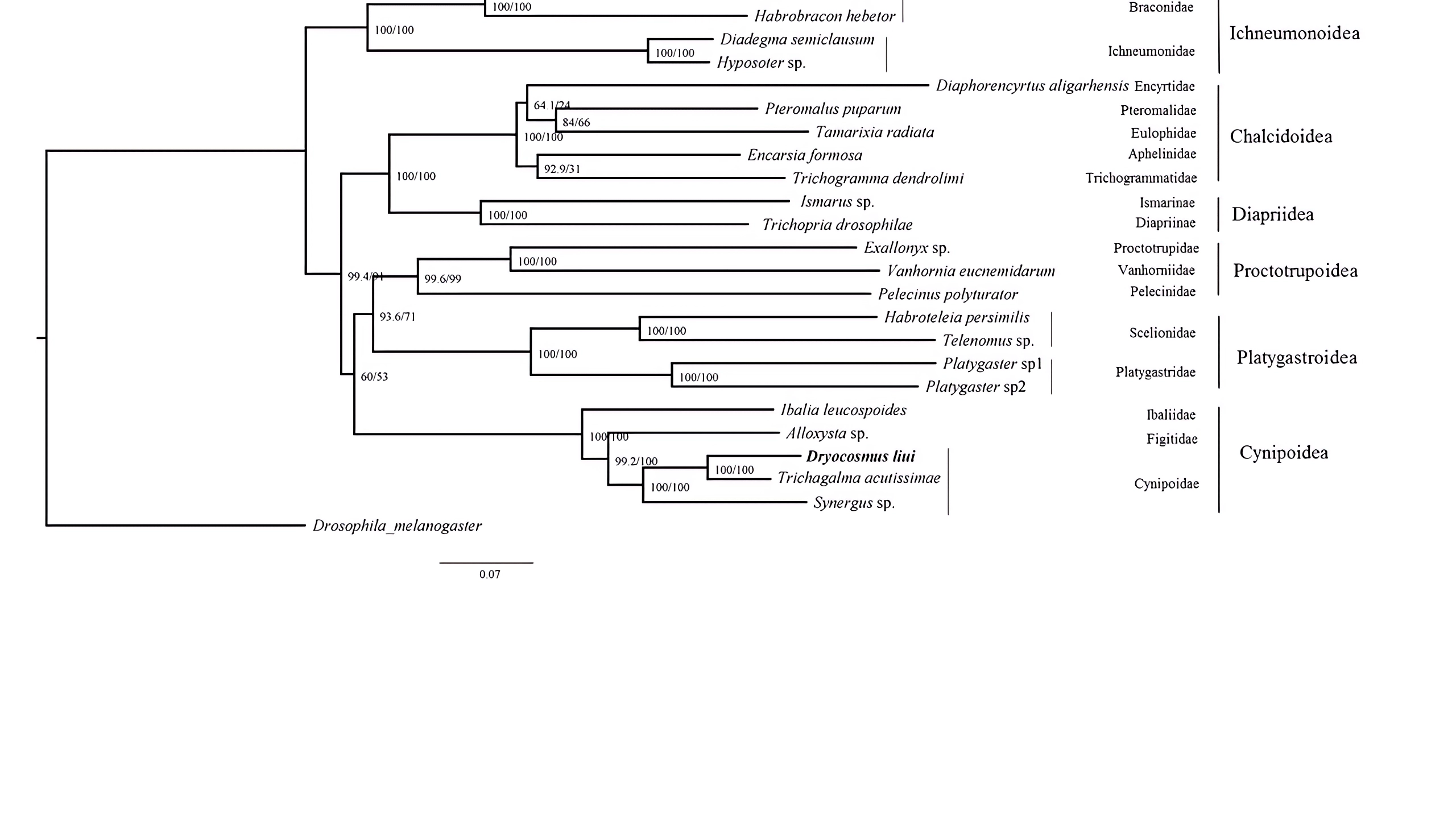

Supplement: Figure S5 — Posterior Probability /approximate likelihood ratio test are shown at each node. [file peerj-11-15865-s005.png]

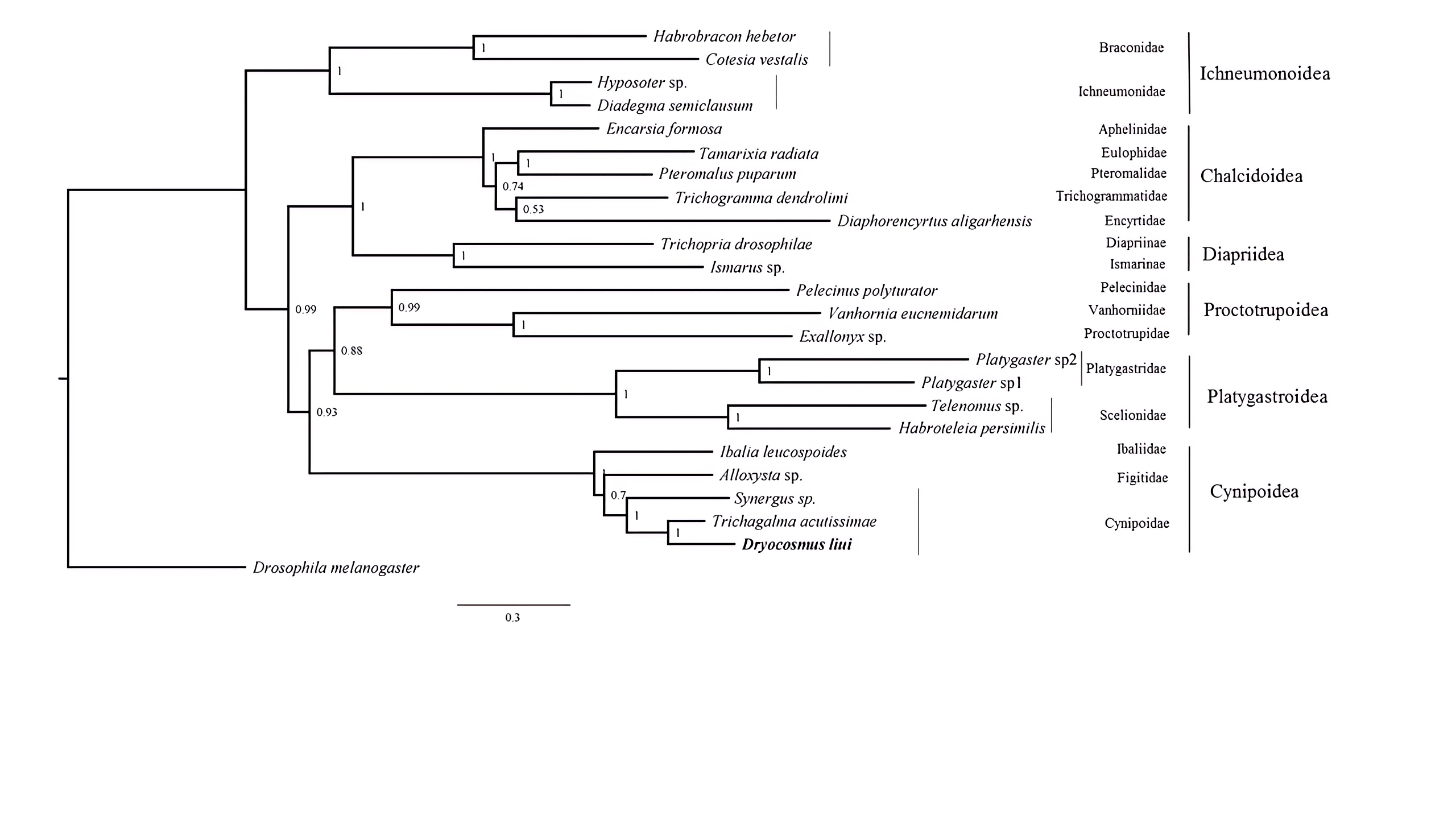

Supplement: Figure S6 — Posterior probabilities are shown at each node. [file peerj-11-15865-s006.png]

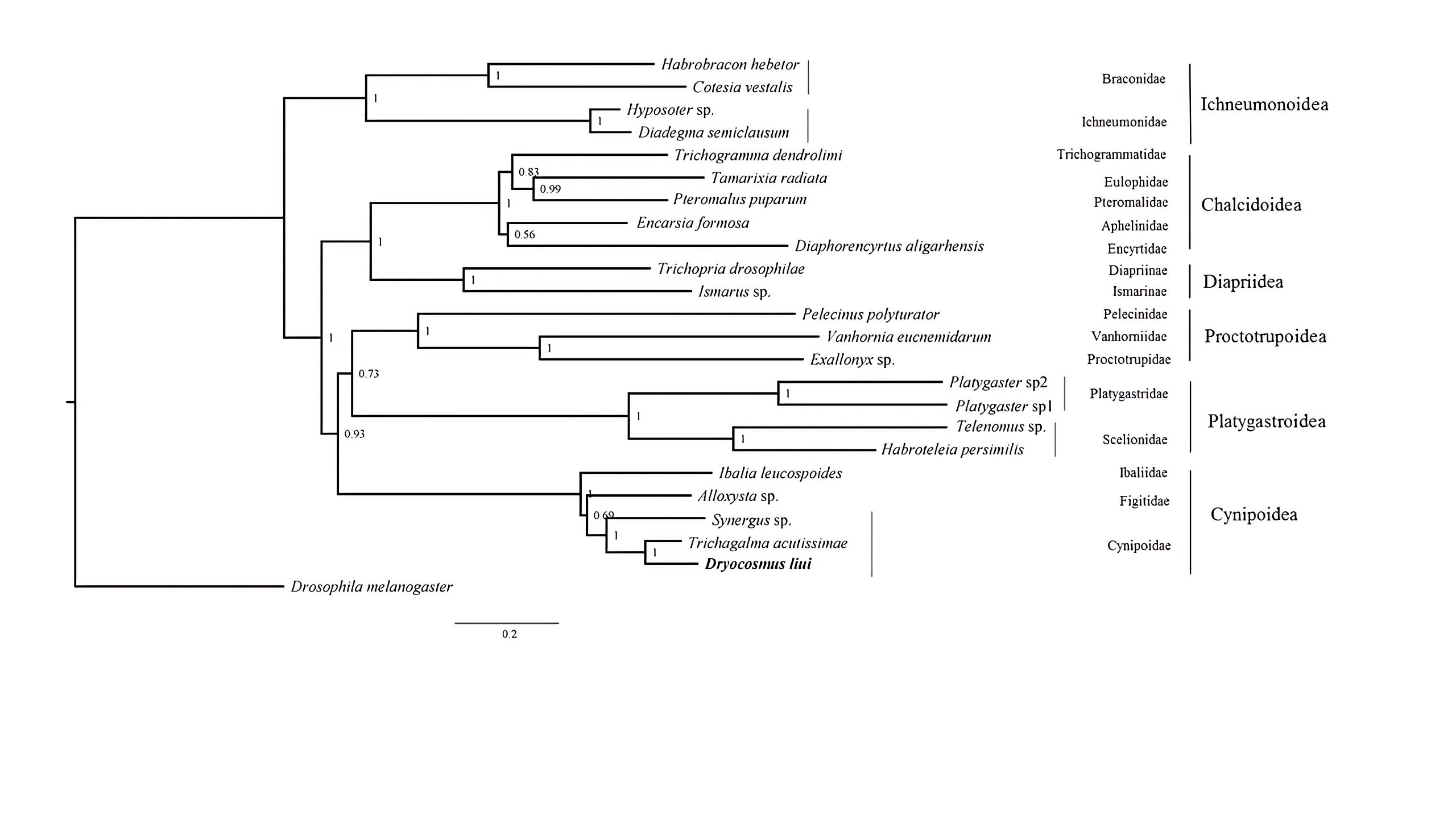

Supplement: Figure S7 — Posterior probabilities are shown at each node. [file peerj-11-15865-s007.png]
