# Supplementary material for: The complete mitochondrial genome and gene rearrangements in a gall wasp species, Dryocosmus liui (Hymenoptera: Cynipoidea: Cynipidae)
Source: PeerJ. 2023 Oct 3;11:e15865. doi: 10.7717/peerj.15865 (PMC10557937; doi:10.7717/peerj.15865)
Supplement: Table S1 [file peerj-11-15865-s008.docx]

Table S1 List of PCR primers for primer developments.

| Name | Locus | Sequence (5'–3') | Reference for primer or sequence |
| --- | --- | --- | --- |
| LCO1490 | cox1 | GGTCAACAAATCATAAAGATATTGG | Folmer et al. (1994) |
| HC02198 | cox1 | TAAACTTCAGGGTGACCAAAAAATCA | Folmer et al. (1994) |
| CB-J-10933 | cob | TATGTACTACCATGAGGACAAATATC | Simon et al. (1994) |
| CB-N-11367 | cob | ATTACACCTCCTAATTTATTAGGAAT | Simon et al. (1994) |
| LR-N13398 | rrnL | CACCTGTTTATCAAAAACAT | Simon et al. (1994) |
| LR-J-12888 | rrnL | TCGATTTGAACTCARATCATGTA | Simon et al. (1994) |
