# Supplementary material for: The complete mitochondrial genome and gene rearrangements in a gall wasp species, Dryocosmus liui (Hymenoptera: Cynipoidea: Cynipidae)
Source: PeerJ. 2023 Oct 3;11:e15865. doi: 10.7717/peerj.15865 (PMC10557937; doi:10.7717/peerj.15865)
Supplement: Table S3 [file peerj-11-15865-s010.docx]

Table S3 List of PCR primers and sequencing primers used in this study.

| Primer name | Sequence (5'–3') | Position in *D.iui* mtDNA  (16819) | Utility |
| --- | --- | --- | --- |
| **DL** **cox1F** | **TATTGATCAAGGAGCAGGGACAGG** | **3101** | **PCR, Sequencing** |
| DL cox1F-F1 | CTCCCTGTTTTAGCTGGAGC | 3345 | Sequencing |
| DL cox 1F-F2 | TTAATAGGGATACCTCGTCG | 4047 | Sequencing |
| DL cox 1F-F3 | TCAATGATATTGAAGGTATG | 4626 | Sequencing |
| DL cox 1F-F4 | TTTTTGATCCCTCAACAAGA | 5323 | Sequencing |
| DL cox 1F-F5 | ATTTTAGAAATTGCTGTATC | 5895 | Sequencing |
| DL cox 1F-F6 | ACTATTAGGCTAGGTGTAAG | 6482 | Sequencing |
| DL cox 1F-F7 | TATGAATGAAAGATAGGGTC | 7160 | Sequencing |
| DL cox 1F-F8 | CTACCGTAATATTCATTCCATC | 7839 | Sequencing |
| DL cox 1F-F9 | TAATACTACTAAGGGAGTTTC | 8399 | Sequencing |
| DL cox 1F-F10 | ATATTACATCTCCAATTCGA | 8950 | Sequencing |
| DL cox 1F-F15 | ATTTTGGATCTTTACTTGGA | 11917 | Sequencing |
| DL cox 1F-F16 | TATGTATTACCATGAGGAC | 12213 | Sequencing |
| DL cobR-F14 | GAAATTATTCCAAATCCAGG | 3515 | Sequencing |
| DL cobR -F13 | TAATGAAAGTGAGCCACAAC | 3886 | Sequencing |
| DL cobR -F5 | TATAGATCTATTAACCATGG | 9596 | Sequencing |
| DL cobR -F4 | TTAAAATTAGGCGGATATGG | 10121 | Sequencing |
| DL cobR -F3 | TTAATAAACATATACTATCA | 10727 | Sequencing |
| DL cobR -F2 | AAACAAGGGGGTGTAGCAC | 11340 | Sequencing |
| DL cobR -F1 | TGCATTATTAATATGAGAGG | 12015 | Sequencing |
| DL cobF-F1 | AATTGGAATAAAACCAATTG | 12833 | Sequencing |
| DL cobF -F2 | TGACGAAATCCCGGTTTCC | 13288 | Sequencing |
| DL cobF -F3 | AATATAAACTTTATCTCCC | 13811 | Sequencing |
| DL cobF -F4 | AAAGTGCCAGCAATAGCGG | 14043 | Sequencing |
| DL cobF -F5 | AGTAAAAATACTGGAAAGTG | 14691 | Sequencing |
| DL cobF -F6 | AAAAATTTTAAGGGATAAGC | 15154 | Sequencing |
| DL 16SR-F6 | TAAAGGATAATAAGCAGATC | 12782 | Sequencing |
| DL 16SR-F5 | AGAACCAAAAAATCACAAGG | 13192 | Sequencing |
| DL 16SR-F4 | AAAGAGGTAGGGAGATAAAG | 13838 | Sequencing |
| DL 16SR-F3 | AAAAGTTTAACCGCTATTGC | 14071 | Sequencing |
| DL 16SR-F2 | ATTCTAAAAACACTTTCCAG | 14721 | Sequencing |
| DL 16SR-F1 | TTTCATTGAGCAGATTAGAC | 15495 | Sequencing |
| **DL 16SR** | **TATTAAGTTCTATAGGGTCTTATCGTC** | **15695** | **PCR, Sequencing** |
| **DL 16SF** | **GCAGTATTTTAACTGTACTAAGGTAGC** | **15523** | **PCR, Sequencing** |
| DL 16SF-F1 | TTACCTTAGGGATAACAGC | 15853 | Sequencing |
| DL 16SF-F2 | ATTATGTTTACAAGAGGAGA | 16544 | Sequencing |
| DL 16SF-F3 | AATTATAGCAAATCCTCCTC | 328 | Sequencing |
| DL 16SF-F4 | TTTATTTGGTCCCTTACGAA | 880 | Sequencing |
| DL 16SF-F5 | TTTTAGTGCAATAACACTC | 1516 | Sequencing |
| DL 16SF-F6 | AAAAGGGAAAAATCCTAC | 2275 | Sequencing |
| DL 16SF-F7 | CTGTTGTAACTGCTCATGC | 2917 | Sequencing |
| DL cox1R-F7 | ATATTAAGTTCTATAGGGTC | 15696 | Sequencing |
| DL cox1R-F6 | AAATAATTAAACCAATTCCTC | 16350 | Sequencing |
| DL cox1R-F3 | AAATTATTTTACAGGTCCCT | 1683 | Sequencing |
| DL cox1R-F2 | ATTCAAAGACTCAGATCTTC | 2397 | Sequencing |
| DL cox1R-F1 | GGAGGATAAACTGTTCATCC | 3142 | Sequencing |
| DL cox1F-F1 | CTCCCTGTTTTAGCTGGAGC | 3345 | Sequencing |
| DL cox1F-F2 | TTAATAGGGATACCTCGTCG | 4047 | Sequencing |
| DL cox1F-F3 | TCAATGATATTGAAGGTATG | 4626 | Sequencing |
| DL cox1F-F4 | TTTTTGATCCCTCAACAAGA | 5323 | Sequencing |
| DL cox1F-F5 | ATTTTAGAAATTGCTGTATC | 5895 | Sequencing |
| DL cox1F-F6 | ACTATTAGGCTAGGTGTAAG | 6482 | Sequencing |
| DL cox1F-F7 | TATGAATGAAAGATAGGGTC | 7160 | Sequencing |
| DL cox1F-F8 | CTACCGTAATATTCATTCCATC | 7839 | Sequencing |
| DL cox1F-F9 | TAATACTACTAAGGGAGTTTC | 8399 | Sequencing |
| DL cox1F-F10 | ATATTACATCTCCAATTCGA | 8950 | Sequencing |
| DL cox1F-F15 | ATTTTGGATCTTTACTTGGA | 11917 | Sequencing |
| DL cox1F-F16 | TATGTATTACCATGAGGAC | 12213 | Sequencing |
| DL cobR-F14 | GAAATTATTCCAAATCCAGG | 3515 | Sequencing |
| DL cobR-F13 | TAATGAAAGTGAGCCACAAC | 3886 | Sequencing |
| DL cobR-F3 | TTAATAAACATATACTATCA | 10727 | Sequencing |
| DL cobR-F2 | AAACAAGGGGGTGTAGCAC | 11340 | Sequencing |
| DL cobR-F1 | TGCATTATTAATATGAGAGG | 12015 | Sequencing |
| **DL cobR** | **GCAAATAGGAAGTATCATTCTGGTTG** | **12654** | **PCR, Sequencing** |
