# Supplementary material for: The complete mitochondrial genome and gene rearrangements in a gall wasp species, Dryocosmus liui (Hymenoptera: Cynipoidea: Cynipidae)
Source: PeerJ. 2023 Oct 3;11:e15865. doi: 10.7717/peerj.15865 (PMC10557937; doi:10.7717/peerj.15865)
Supplement: Table S4 [file peerj-11-15865-s011.docx]

Table S4 Summary of taxonomic groups used in this study.

| **Sequence Name** | **Superfamily** | **Family** | **Accession Number** | **Reference** |
| --- | --- | --- | --- | --- |
| *Alloxysta* sp. | Cynipoidea | Figitidae | MG923482 | Tang et al. 2019 |
| *Cotesia vestalis* | Ichneumonoidea | Braconidae | FJ154897 | Wei et al. 2010 |
| ***Dryocosmus liui*** | **Cynipoidea** | **Cynipidae** | **MW368384** | **this study** |
| *Diadegma semiclausum* | Ichneumonoidea | Ichneumonidae | EU871947 | Wei et al. 2009 |
| *Diaphorencyrtus aligarhensis* | Chalcidoidea | Encyrtidae | NC046058 | Du et al. 2019 |
| *Exallonyx* sp. | Proctotrupoidea | Proctotrupidae | MG923495 | Tang et al. 2019 |
| *Encarsia formosa* | Chalcidoidea | Aphelinidae | MG813797 | Zhu et al. 2018 |
| *Hyposoter* sp. | Ichneumonoidea | Ichneumonidae | MG923499 | Tang et al. 2019 |
| *Habrobracon hebetor* | Ichneumonoidea | Braconidae | MN842279 | Huang et al. 2020 |
| *Habroteleia persimilis* | Platygastroidea | Scelionidae | MG923508 | Tang et al. 2019 |
| *Ibalia leucospoides* | Cynipoidea | Ibaliidae | KJ814197 | Mao et al. 2015 |
| *Ismarus* sp. | Proctotrupoidea | Diapriidae | MG923501 | Tang et al. 2019 |
| *Platygaster* sp1 | Platygastroidea | Platygastridae | MG923510 | Tang et al. 2019 |
| *Pteromalus puparum* | Chalcidoidea | Pteromalidae | NC039656 | unpublished |
| *Pelecinus polyturator* | Proctotrupoidea | Pelecinidae | NC026865 | Mao et al. 2015 |
| *Platygaster* sp2 | Platygastroidea | Platygastridae | MG923507 | Tang et al. 2019 |
| *Synergus* sp. | Cynipoidea | Cynipidae | MG923514 | Tang et al. 2019 |
| *Tamarixia radiata* | Chalcidoidea | Eulophidae | MN123622 | Du et al. 2019 |
| *Trichagalma acutissimae* | Cynipoidea | Cynipidae | MN928529 | Xue et al. 2020 |
| *Trichopria drosophilae* | Proctotrupoidea | Diapriidae | MN966974 | Zhang et al. 2020 |
| *Telenomus* sp. | Platygastroidea | Scelionidae | MF776884 | Shen et al. 2019 |
| *Trichogramma dendrolimi* | Chalcidoidea | Trichogrammatidae | KU836507 | unpublished |
| *Vanhornia eucnemidarum* | Proctotrupoidea | Vanhorniidae | DQ302100 | Castro et al., 2006 |

References

Castro L.R; Ruberu K; Dowton M. Mitochondrial genomes of Vanhornia eucnemidarum (Apocrita: Vanhorniidae) and *Primeuchroeus* sp. (Aculeata: Chrysididae): evidence of re-arranged mitochondrial genomes within the Apocrita (Insecta: Hymenoptera). Genome 2006, 49 (7), 752-766.

Du Y.M; Song X.;Liu X.J;Ouyang Z.G; Lu Z.J. Mitochondrial genome of *Tamarixia radiata* (Hymenoptera:Chalcidoidea: Eulophidae) and phylogenetic analysis. Mitochondrial DNA B. 2019, 4 (2), 2839-2840.

Du Y.M; Song X.;Liu X.J; Zhong B.L. Mitochondrial genome of *Diaphorencyrtus aligarhensis* (Hymenoptera: Chalcidoidea: Encyrtidae) and phylogenetic analysis. Mitochondrial DNA B. 2019, 4 (2), 3190-3191.

Huang Y.X; Qi L.Q; Zhang Y.Z; Jin X.X; Wang X. Sequencing and analysis of the complete mitochondrial genome of *Habrobracon hebetor* (Hymenoptera: Braconidae). Mitochondrial DNA B. 2020, 5 (1), 1009-1010.

Wei S.J; Shi M.; He J.H; Sharkey M.; Chen X.X. The complete mitochondrial genome of *Diadegma semiclausum* (hymenoptera: ichneumonidae) indicates extensive independent evolutionary events. Genome 2009, 2 (4), 308-319.

Wei S.J; Shi M; Sharkey M.J; [Achterberg](https://www.ncbi.nlm.nih.gov/pubmed/?term=van%20Achterberg%20C%5BAuthor%5D&cauthor=true&cauthor_uid=20537196) C; [Chen](https://www.ncbi.nlm.nih.gov/pubmed/?term=Chen%20Xx%5BAuthor%5D&cauthor=true&cauthor_uid=20537196) X.X. Comparative mitogenomics of Braconidae (Insecta: Hymenoptera) and the phylogenetic utility of mitochondrial genomes with special reference to Holometabolous insects. BMC Genomics. 2010, 11, 371.

Shen Z.C; Chen L.; Chen L.; Li Y.X; Information from the mitochondrial genomes of two egg parasitoids, *Gonatocerus* sp. and *Telenomus* sp., reveals a controversial phylogenetic relationship between Mymaridae and Scelionidae. Genomics. 2019, 111 (5), 1059-1065.

Zhu, J.C.; Tang, P.; Zheng, B.Y.; Wu, Q.; Wei, S.J.; Chen , X.X. The first two mitochondrial genomes of the family Aphelinidae with novel gene orders and phylogenetic Implications. Int J Biol Macromol. 2018, 118, 386-396.

Zhang, X.; Pan, Z.Q.; Chen, J.N.; Zhu, J.C.; Zhou, S.C.; Pang, L.; Shi, M.; Chen, X.X.; Huang, J.H. The complete mitochondrial genome of *Trichopria drosophilae* (Hymenoptera: Diapriidae). Mitochondrial DNA B.2020,  5 (3), 2391-2393.
