# Supplementary material for: The complete mitochondrial genome and gene rearrangements in a gall wasp species, Dryocosmus liui (Hymenoptera: Cynipoidea: Cynipidae)
Source: PeerJ. 2023 Oct 3;11:e15865. doi: 10.7717/peerj.15865 (PMC10557937; doi:10.7717/peerj.15865)
Supplement: Table S5 [file peerj-11-15865-s012.docx]

Table S5 Genome feature analysis.

| Base composition | A | T | G | C | A+T% | AT-skews | GC-skews |
| --- | --- | --- | --- | --- | --- | --- | --- |
| Total | 7053 | 7030 | 1355 | 1381 | 83.7 | 0.0016 | -0.0095 |
| *trn* | 656 | 653 | 83 | 96 | 88.0 | 0.0023 | -0.0726 |
| PCG | 4467 | 4521 | 977 | 1033 | 81.7 | -0.0060 | -0.0279 |
| *rrn* | 986 | 871 | 137 | 86 | 89.3 | 0.0619 | 0.2287 |
| CR | 689 | 722 | 142 | 151 | 82.8 | -0.0234 | -0.0307 |
